# 

**Published:** 1908-05-16

**Authors:** 


CONTENTS.

| | PAGE |
|---|---|
| **Radiography and the Early Diagnosis of Phthisis** | 163 |
| **"Confessio Medici"** ... ... ... ... ... ... ... | 164 |
| **Annotations**— | |
| Liability for a Consultant's Fee—Territorial General Hospitals —Hospital or Infirmary? ... ... ... ... ... ... | 165 |
| **Medical Opinion and Movement** ... ... ... ... | 166 |
| **Hospital Clinics**— | |
| On the Management and Treatment of Hæmoptysis. By William MacLennan, M.B. ... ... ... ... ... | 169 |
| **Special Article**— | |
| Some Conditions under which Infants are Nursed away from Home ... ... ... ... ... ... ... ... ... | 171 |
| **Clinical Points**— | |
| Functional or Hysterical Pyrexia ... ... ... ... | 175 |
| Reducing Substances in the Urines of Alcoholics ... ... | 176 |
| **Points in Surgery**— | |
| The Surgical Significance of Vomiting.—II.... ... ... | 177 |
| **Mental Diseases**— | |
| Hallucinations ... ... ... ... ... ... ... ... | 178 |
| **Laryngology and Rhinology**— | |
| Syphilis of the Throat and Nose.—II.... ... ... ... ... | 179 |
| **Public Health and Hygiene**— | |
| The Control of Infantile Mortality ... ... ... ... | 180 |
| **Dietetic Notes** ... ... ... ... ... ... ... ... | 180 |
| **Resident Medical Officers' Department**— | |
| Some Medico-Legal Points ... ... ... ... ... | 181 |
| **The General Practitioner's Column**— | |
| The Diagnosis and Significance of Enlarged Mediastinal Glands | 182 |
| **Hospital Administration**— | |
| Graduate Study on the Continent.—Germany ... ... ... | 183 |
| Social and Poor-law Problems ... ... ... ... ... | 184 |
| New Appliances and Things Medical ... ... ... ... | 184 |
| News and Coming Events ... ... ... ... ... | 185 |
| **Editor's Letter-Box** ... ... ... ... ... ... ... | 186 |
| **Nursing Administration**— | |
| The Bedding and Linen Department.—II. ... ... ... ... | 187 |
| **The Graduates' Medical Schools** ... ... ... ... | 188 |

